# Harnessing the Induction of CD8^+^ T-Cell Responses Through Metabolic Regulation by Pathogen-Recognition-Receptor Triggering in Antigen Presenting Cells

**DOI:** 10.3389/fimmu.2018.02372

**Published:** 2018-10-25

**Authors:** Francesco Nicoli, Stéphane Paul, Victor Appay

**Affiliations:** ^1^Department of Molecular Medicine, University of Padua, Padua, Italy; ^2^GIMAP/EA3064, Université de Lyon, CIC 1408 Vaccinology, Saint-Etienne, France; ^3^Sorbonne Université, INSERM, Centre d'Immunologie et des Maladies Infectieuses, Paris, France; ^4^International Research Center of Medical Sciences, Kumamoto University, Kumamoto, Japan

**Keywords:** immunometabolism, pathogen-recognition-receptor, TLR4, TLR9, STING, adjuvants, CD8^+^ T-cell priming

## Abstract

Cytotoxic CD8^+^ T-cells are key players of the immune responses against viruses. During the priming of a CD8^+^ T-cell response, the activation of a naïve T-cell by a professional antigen presenting cell (APC) involves the induction of various intracellular and metabolic pathways. The modulation of these pathways at the level of APCs or T-cells offers great potential to enhance the induction of robust effector cells and the generation of long-lived memory cells. On the one hand, signaling through pathogen recognition receptors (PRRs) expressed by APCs can greatly influence T-cell priming, and the potential of several PRR ligands as adjuvants are being studied. On the other hand, the engagement of several metabolic processes, at play in APCs and T-cells upon stimulation, implies that modulating cellular metabolism can impact on priming efficacy. Here, we review recent efforts to understand the interplay between PRR mediated signaling and metabolic pathway modulation in this context, through three examples: interplay between TLR4 and fatty acid metabolism, between TLR9 and IDO, and between STING and autophagy. These initial works highlight the potential for harnessing the induction of antiviral CD8^+^ T-cell responses using synergistic modulation of metabolic and PRR pathways.

## Introduction

CD8^+^ T-cells are major actors of the fight against viruses. Owing to their capacity, through T-cell receptor (TCR)—peptide Major Histocompatibility Complex (pMHC) interactions, to recognize a diversity of antigens presented on virus infected cells, CD8^+^ T-cells can directly kill target cells. However, rather than their quantity or frequency, their quality, or aptitude to engage multiple effector functions, represents an important basis of their efficacy in viral infection settings (1, 2). Induction of CD8^+^ T-cells with superior qualitative properties is therefore a primary goal of vaccines and immunotherapies in this context. The acquisition of functional attributes by CD8^+^ T-cells is crucially dependent on the priming step of the response, when antigen specific naïve precursors get activated and expand in response to the presentation of their cognate antigen by dendritic cells (DCs) (3, 4). In recent years, we have gained increasing knowledge about the determinants of the quality of CD8^+^ T-cells, and how to influence them upon priming. For instance, dendritic cells (DCs) govern the nature of primed CD8^+^ T-cells via the provision of processed antigens in the form of pMHC class I molecules (signal I) and other important signals, including costimulatory interactions (signal II) and inflammatory cytokines (signal III) (5). Much effort has been focused on the modulation of DC function through pathogen-recognition-receptor (PRR) triggering (6, 7), as PRR ligands can modulate these different signals and thereby enhance the priming process to elicit more robust T-cell responses (7–9). Molecules, such as Toll-like receptor (TLR) ligands, can improve the immunogenicity of antigens by mimicking pathogen-associated “danger” signals in order to improve T-cell immunity (10, 11).

Moreover, new insights into cellular metabolism have underlined the tight connection existing between metabolic and functional properties of immune cells (12). For instance, recent studies have demonstrated that aerobic or catabolic metabolic processes and mitochondrial biogenesis control CD8^+^ T-cell effector and memory cell formation (13, 14). In response to activation, CD8^+^ T-cells undergo a metabolic transition or reprogramming. Quiescent naive T-cells have a low metabolic demand and rely primarily on oxidative phosphorylation (OXPHOS) (15, 16). Upon activation though, they switch to a AKT/mTOR-orchestrated reliance on multiple metabolic pathways including aerobic glycolysis, glutaminolysis and OXPHOS, which are important for the acquisition of effector functions and sustained proliferation (15–18). Eventually, memory CD8^+^ T-cells regain a more catabolic metabolism and preferentially rely on fatty acid (FA) synthesis to fuel FA oxidation and enhance mitochondria respiratory capacity, and thus provide survival advantages (19). Cellular metabolic intermediates are therefore major regulators of CD8^+^ T-cell activation and can dictate functional performance of effector cells upon priming (20). This opens new avenues to modulate cellular metabolic activity in order to promote the induction of high quality immune responses and enhance antiviral as well as antitumor CD8^+^ T-cell immunity. In this review, we discuss initial considerations regarding the metabolic parallels between PRR- or TCR-mediated stimulation, and recent works highlighting how the quality of primed CD8^+^ T-cells may be altered through metabolic regulation of T-cells or DCs using PRR agonists.

## Differences and similarities between PRR- and TCR-induced metabolic reprogramming

The activation of both APCs via PRRs and T-cells via TCR is energetically reliant on the adoption of anabolic processes, and in particular on the consumption of glucose and production of lactate by a metabolic pathway called Warburg metabolism or aerobic glycolysis (15, 21–27). The rapid engagement of glycolysis has been shown in response to a broad array of PRR agonists, including ligands for TLR2, 4, 7, 9, and C-type lectin receptors, and is essential to support their stimulatory effects (22–25). Similarly, glycolysis is required for differentiation into effector cells and cytokine secretion in T lymphocytes upon TCR-mediate activation (26, 27).

The anabolic processes that regulate the activation of both DCs and T-cells are under the control of mTOR (15, 21), which is essential for differentiation of T-cells (28, 29) as well as for the maturation, differentiation, survival and T-cell stimulatory activity of DCs (21, 30–34). The glycolytic burst occurring in APC and T-cell upon activation is also supported by mTOR, via the transcription factors Hypoxia-inducible factor-1α (HIF-1α), that prompts the expression of key glycolytic enzymes (35–37). However, it has been reported that TCR-induced proliferation may occur also in the presence of mTOR inhibition (28, 29), which instead improves pro-inflammatory effects of TLR stimulation, resulting in enhanced IL-12 production and reduced IL-10 release by DCs (33, 38, 39), depending on the DC type (33). Therefore, the exact involvement of mTOR in integrating TCR and PRR signaling is not completely understood, and clues indicate a different role for this kinase in DC and T-cell activation.

Of note, TLR-induced metabolic reprogramming involves also the activation of *de novo* fatty acid synthesis (FAS) (23), required for the production of membranes to expand organelles (23). Interestingly, FAS is induced also after T-cell activation, and necessary for their expansion (12, 40). The induction of FAS upon PRR and TCR stimulation leads to the storage of fatty acids in lipid droplets (23, 41), whose function still remains controversial. Indeed, DCs with high content of lipids have been shown to better activate T-cells in the liver (42) but displayed diminished priming capacity within tumors (43). In addition, while storage of FA into triacylglycerol may be a mechanism exerted to avoid lipotoxicity (44), excess on neutral lipids has also been shown to induce apoptosis in T-cells (45).

## Interplay between TLR4 and fatty acid metabolism

The canonical Toll-like receptor 4 (TLR4) signaling cascade is initiated when lipid A (the membrane anchor of lipopolysaccharide [LPS]) is bound by the extracellular region of CD14, which complexes with MD2 and binds to membrane-bound TLR4 (46). Dimerization of these molecules with another lipid A-MD2-TLR4 complex creates a functional TLR4 signaling complex (47). Binding of a TLR4 agonist like lipid A initiates an innate immune response that can drive the development of antigen-specific acquired immunity (48). Mimicking the innate sensing of molecular patterns derived from microbes—pathogenic and non-pathogenic—to activate of immune cells, TLR4 agonist molecules show great promise for use as immunotherapeutic adjuvants to potentiate host responses in component vaccines [Reviewed in Reed et al. (48)].

**Figure 1:**
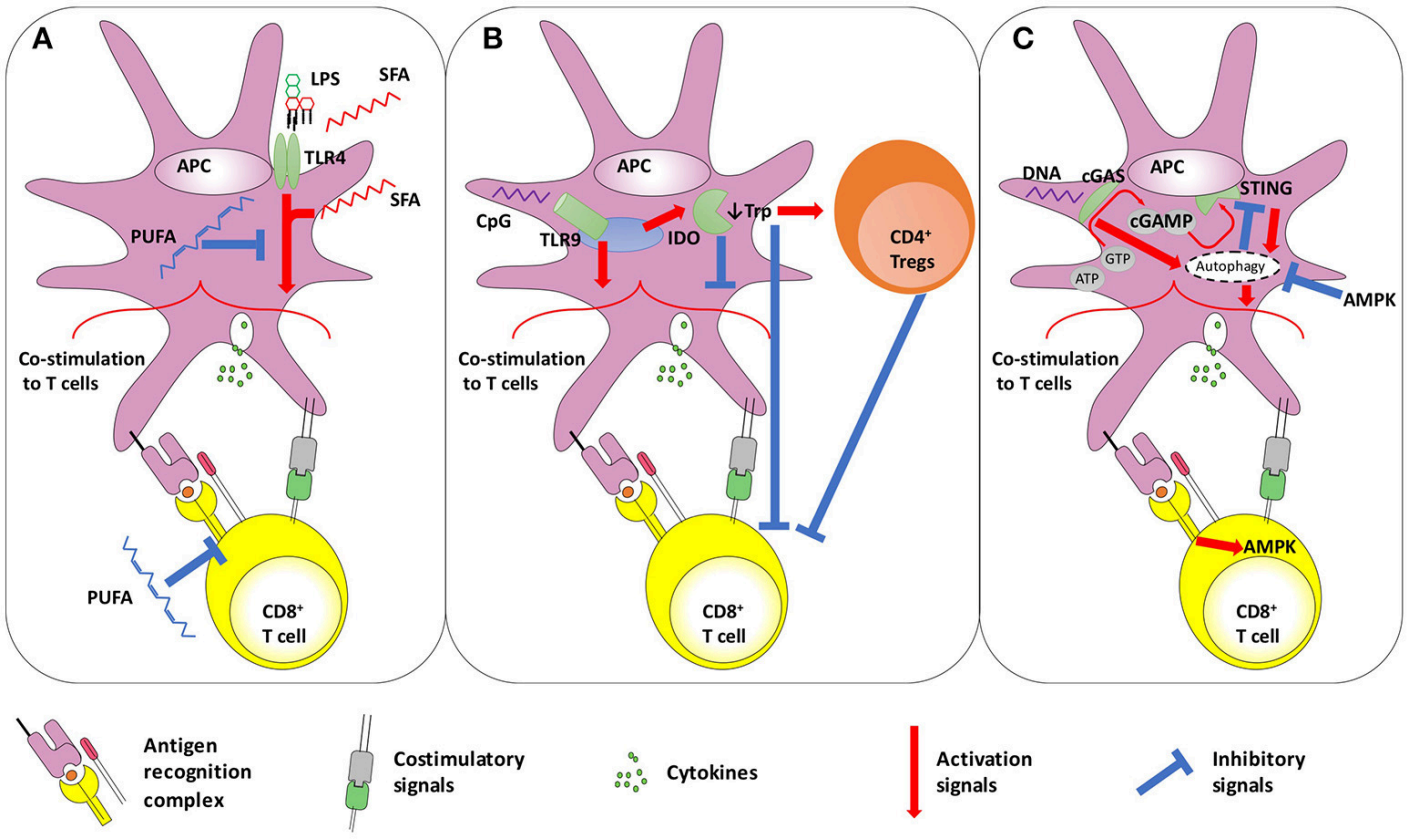
Schematic representation of the interplay between **(A)** TLR4 and fatty acid metabolism, **(B)** TLR9 and IDO, and **(C)** STING and autophagy. **(A)** TLR4 activation on APCs improves CD8^+^ T-cell priming. In addition to LPS, SFA are also thought to trigger TLR4. However, it has also been proposed that SFA act on TLR4-downstream pathways. In contrast, PUFA display anti-inflammatory effects, by dampening both TLR4- and TCR-induced signaling. **(B)** Dual role of TLR9 stimulation on T-cell activation. The TLR9 ligand CpG shows adjuvant effects, improving the co-stimulation delivered by APCs to T-cells. However, some reports highlighted that the same pathway may also trigger negative regulators of immunity, such as IDO that down-modulates APC-provided co-stimulation and favors Treg activity. Furthermore, IDO mediates tryptophan deprivation, with has negative consequences on T-cell functionality. **(C)** The autophagy-STING loop. The cytosolic DNA sensors cGAS converts ATP and GTP into the dinucleotide cGAMP, which triggers STING. Both cGAS and STING may promote authophagy, that can be involved in two distinct processes: inducing APC-delivered co-stimulation to T-cells, and STING degradation to avoid its permanent activation. The latter process seems under the control of AMPK, a kinase also acting in downstream TCR signaling in T-cells. AMPK, AMP-activated protein kinase; APC, antigen presenting cell; ATP, Adenosine Triphosphate; cGAMP, cyclic guanosine monophosphate–adenosine monophosphate; cGAS, cGAMP synthase; CpG, CpG oligodeoxynucleotides; GTP, Guanosine Triphosphate; IDO, Indoleamine 2;3-dioxygenase; Trp, tryptophan; LPS, lipopolysaccharide; PUFA, poly-unsaturated fatty acids; SFA, saturated fatty acids; STING, stimulator of interferon genes; TLR, toll like receptor.

With respect to metabolism, TLR4 stimulation has been linked with FA-induced inflammation in a number of pathologic conditions, including insulin resistance, retinal impairment, atherosclerosis and myocardial injury observed during diabetes and obesity (49–54). Long chain, saturated FAs (SFAs) require TLR4 to exert pro-inflammatory effects (55), and have been suggested to bind it (53, 56). Lipid A itself is acylated with SFAs (57), whose number, length and saturation determine the TLR4 agonistic properties of LPS (49, 57). Conversely, poly-unsaturated FAs (PUFAs) inhibit TLR4 activation (49, 58). Notably, a similar pattern has been shown for another bacterial cell wall sensor, TLR2 (59). More recently, it has been proposed that SFAs may act as agonists of TLR4 without binding it (55, 60). SFAs may indeed be able to induce TLR4 dimerization in lipid rafts, in a ligand-independent manner (61), a step that is inhibited by PUFA. Irrespective of the mechanisms, evidence is concordant in suggesting that saturated and polyunsaturated FAs exert opposite effects on TLR4-mediated inflammatory response and APC activation. Indeed, SFAs may up-regulate the expression of costimulatory molecules and cytokines, resulting in increased T-cell activation capacity, while these effects are inhibited by PUFA (62). Several lines of evidence suggest that PUFA may reduce the induction of T-cell responses (63–65), acting on both APCs and T-cells. In addition to preventing TLR4 dimerization in lipid rafts and inhibiting downstream kinases (61, 66), PUFA can affect lipid rafts composition in T-cells, altering TCR signaling (67, 68) and resulting in hampered T-cell functionality (68–70). Overall, SFAs may favor co-stimulation delivered by APCs to T-cells and favor both TLR4 and TCR signaling (71), thus potentially boosting priming capacity (Figure 1A).

However, the role of specific FA species on T-cell functionality is not yet completely understood (44). Although it appears clear that FA are required during T-cell expansion (72), their excess may result in reduced T-cell proliferation and increases apoptosis (44, 73), and their use as energy source (fatty acid oxidation—FAO) was initially considered not to contribute to T-cell expansion following priming (74), although important for the transition of primed T-cells toward memory (74). Conversely, it has been recently discovered that FAO may sustain metabolic shift occurring upon TLR4 and TCR stimulation, in low glucose concentration conditions (75, 76) and during graft-vs.-host disease (77), suggesting a potential role for FAO in T-cell priming. SFA-mediated pro-inflammatory signaling requires their ligation with coenzyme A, a necessary step for SFA oxidation (55), indicating that FAO may be important to facilitate pro-inflammatory effects. This indicates therefore that the enhancement of FA catabolism may synergize with TLR4 activation to boost T-cell priming. Although further studies are necessary to better understand the underlying mechanisms, three hypotheses about the role of FAO in boosting T-cell priming may be proposed: (i) the induction of pro-inflammatory signals; (ii) the provision of additional energy sources to the activated APCs and T-cells, and (iii) the removal of high (and potentially toxic) concentration of SFAs or of FA with inhibitory activity (such as PUFA).

## Interplay between TLR9 and IDO

TLR9 is an endosomal receptor recognizing specific unmethylated CpG motifs present at high frequency in bacterial genome but absent in the mammalian one. TLR9 signals via the adaptor protein MyD88, leading to the production of pro-inflammatory cytokines (after activation of the NF-κB pathway) and type I interferon (after activation of the IRF7 pathway) (78–80). Interestingly, TLR9 has also been identified as a specific sensor of RNA:DNA hybrids, a key intermediate component essential to the replication during infection. The use of TLR9 agonists as vaccine adjuvant presents a great potential [Reviewed in Scheiermann and Klinman (81)], and DNA vaccines containing unmethylated CpG motifs show an enhanced immunogenicity (7, 82).

Nonetheless, increasing evidence indicates that TLR9 stimulation may also have immunosuppressive/tolerogenic effects. Despite the lack of consensus on this issue, the major mechanism explaining this phenomenon is the TLR9-mediated modulation of Indoleamine 2,3-dioxygenase (IDO), that catalyzes the first step of tryptophan catabolism (Figure 1B). *In vivo* systemic treatment with different TLR9-ligands could decrease the onset/severity of autoimmune diseases but increase susceptibility to infections in a IDO-dependent manner (83–87). Indeed, high CpG oligodeoxynucleotide doses may induce IDO in pDCs and splenocytes (86, 88), reducing the secretion of pro-inflammatory cytokines and favoring the expression of PD-L1, fostering the acquisition of suppressive activity by Tregs (89) and reducing antigen-specific T-cell expansion (86, 88, 90). Nonetheless, TLR9-mediated IDO induction of immunosuppressive properties depends on the type of TLR9 ligand used, as well as on the dose and route of administration (85–87, 90). The induction of IDO expression is a well-known immunosuppressive mechanism, which is also observed in several viral infections (91, 92). In addition to TLRs, IDO expression may also be induced upon stimulation of several receptors, including those for type I and II interferons, CD40L and TGFβ (93). Tryptophan degradation in the kynurenine pathway (KP), whose first step is mediated by IDO, may lower the concentrations of this amino acid, essential for cell survival and proliferation, and result in the synthesis of KP metabolites with immunosuppressive activity (93). Tryptophan depletion inhibits mTORC1 activity in T-cells as well as their proliferation (93, 94), while moDCs and pDCs expressing IDOs might prompt Treg expansion and suppressive activity (95, 96). As a result, T-cell priming efficacy and the generation of robust antiviral and memory responses was shown to be ameliorated by the use of IDO inhibitors *in vivo* (97–99). The use of IDO inhibitors may therefore reduce immunosuppressive effects of TLR9 ligands and boost its adjuvant activity, favoring the induction of strong antiviral and antitumor T-cell responses (Figure 1B).

## Interplay between STING and autophagy

In the recent years, a strong enthusiasm for the study of the stimulator of interferon genes (STING) pathway has led to a better knowledge of the complexity of cytosolic DNA sensors (100). First identified as an adaptor protein mediating innate immune signaling induced by cytosolic DNA sensors, STING's function as cyclic di-nucleotide sensor has been described only recently (101), generating great enthusiasm for its potential use in cancer immunotherapy [Reviewed in Iurescia et al. (102)]. STING is a receptor for cyclic guanosine monophosphate–adenosine monophosphate (cGAMP), which can be synthesized by cGAS (cGAMP synthase), a member of the nucleotidyl transferase family. The latter plays a role in the recognition of HIV and other retroviruses leading to the synthesis of cGAMP (103). The produced cGAMP acts as an endogenous second messenger that binds to STING, leading to the activation of IRF3 and the induction of type I interferon synthesis (101). In addition to its major role for RNA virus sensing, it has been shown that cGAS expression also broadly inhibits several DNA viruses. However, the effect of cGAS is not limited to viruses. It acts as a DNA sensor responsible for the recognition of *Mycobacterium tuberculosis*, leading to the activation of the STING pathway. The recognition of this bacteria, as well as others, is made through cyclic-di-AMP, a bacterial cyclic di-nucleotide (CDN) leading to the production of IFN-β. CDN are also able to stimulate STING directly, and to activate an innate immune response leading to the induction of type I interferons. Interestingly, it has been demonstrated that RNA:DNA hybrids are also sensed by the immune system through the cGAS-STING pathway, inducing a strong type I interferon response. cGAMP has proven to be an effective adjuvant, able to boost the production of antigen-specific antibodies and T-cell responses after an intramuscular administration in mice. It has been recently evidenced that cGAMP is a promising mucosal adjuvant. STING agonists are also novel and highly promising immunomodulators for cancer immunotherapy (104). Its activation by CDN has proved to be efficient for anti-tumoral vaccination against metastatic breast cancer. Surprisingly, the STING pathway can also be triggered upon mitochondrial damage through the generation of mitochondrial ROS and the release of endogenous DNA into the cytosol.

Increasing evidence indicates that pathogen-derived CDN may trigger autophagy via STING (105–107), which forms cytoplasmic structures with LC3 and Atg9a, two proteins involved in the autophagy process (108). However, controversies exist about the significance of STING-induced autophagy. Indeed, STING and TBK1 migrates together via an autophagy-like process (109), and autophagy inhibition in cells infected with viruses known to activate STING dampens type I interferon production (107, 109). This suggests that autophagy seems essential to STING mediated pro-inflammatory effects (Figure 1C). Consistently, downstream STING type I IFN induction is dependent on Vps34 (110), a phosphatidylinositol 3-kinase (PI3K) required for autophagy initiation (111). However, other two important autophagy-related proteins, Beclin-1 and the serine/threonine protein kinase ULK1, are dispensable for STING pro-inflammatory effects (110) but, rather, involved in STING degradation. ULK1 is activated, after the formation of STING-dependent autophagosomes, by the same cyclic dinucleotides that activate STING, but mediates its phosphorylation and blocking (110), while Beclin-1 interacts with cGAS to promote autophagy in a STING-independent manner, dampening interferon responses (112). Thus, autophagy would prompt STING degradation to avoid its chronic activity (110, 113). The dual role of autophagy in STING stimulation, delivering STING pro-inflammatory signaling at first and then mediating its degradation, suggests a temporal biphasic function of this metabolic process. Interestingly, a similar pattern has been described in T-cell activation: autophagy has been shown to first support NF-κB signaling in T-cells to then downregulate it (114, 115). Autophagy is activated and needed at the beginning of TCR stimulation to sense, and thus activate, mTOR (116, 117); then mTOR itself shuts down autophagy, which seems no longer required for effector cell generation, although essential for memory cell formation (117–119).

Further studies are needed to investigate which autophagy-related proteins should be targeted to improve STING adjuvant effects, enhancing downstream signaling and postponing its degradation to ensure prolonged STING activity at least during the initial phases of T-cell priming. Notably, cGAS-Beclin-1 mediated STING regulation is prompted by ligands, but not products of cGAS (such as 2′3′cGAMP), suggesting that the use of direct STING agonists may overcome this control mechanism leaving unaffected STING-induced autophagy. In addition, as the ULK1-dependent negative feedback is regulated by AMPK (110), whose inhibition leads to ULK1 activation, STING degradation and type I IFN response reduction (110, 120), AMPK activators might be used to prolong STING activity. It should be noted that AMPK has often been considered as anti-inflammatory, also for its capacity to suppress mTOR activity (121), which is required for T-cell activation. Nevertheless, AMPK activation occurs during and is essential for primary T-cell responses (74, 121, 122), may boost the generation of memory cells (74, 121), restore the functionality of exhausted effector cells (123) and generate robust effector cells starting from naïve cells (121, 123). Therefore, the potential use of AMPK activators in combination with STING ligands for priming of T-cell responses should be further explored with the aim to prolong STING pro-inflammatory activity, counteract exhaustion and prompt the generation of the memory pool.

## Concluding remarks

The discovery of PRRs and their ligands certainly represents one of the most fundamental advances of modern immunology with many, some yet to discover, applications in the context of vaccine development. In the recent years, our growing perception of the importance of immunometabolism is also opening new directions for immune interventions. Although it is still early days, the examples discussed in the present review provide clear evidence that combining our knowledge on metabolic immune regulation and PRR pathway activation offer great potential to influence the induction of potent immune responses. It will be important to assess the prospective use of such therapeutic approaches in animal or pre-clinical studies in order to better characterize benefits and drawbacks of these strategies in *in vivo* settings. Eventually, the combination of metabolic regulators and PRR based adjuvants may prove particularly effective in context of difficult to vaccinate populations, such as the elderly, whom immune cells present both metabolic and functional alterations, and overall suboptimal immune responsiveness.

## Author contributions

FN, SP, and VA reviewed the literature and wrote the manuscript.

### Conflict of interest statement

The authors declare that the research was conducted in the absence of any commercial or financial relationships that could be construed as a potential conflict of interest.

## References

[1] Nicoli F Gallerani E Skarlis C Sicurella M Cafaro A Ensoli B. Systemic immunodominant CD8 responses with an effector-like phenotype are induced by intravaginal immunization with attenuated HSV vectors expressing HIV Tat and mediate protection against HSV infection. Vaccine (2016) 34:2216–24. 10.1016/j.vaccine.2016.03.022 27002499

[2] Almeida JR Price DA Papagno L Arkoub ZA Sauce D Bornstein E. Superior control of HIV-1 replication by CD8+ T cells is reflected by their avidity, polyfunctionality, and clonal turnover. J Exp Med. (2007) 204:2473–85. 10.1084/jem.20070784 17893201 PMC2118466

[3] Mercado R Vijh S Allen SE Kerksiek K Pilip IM Pamer EG. Early programming of T cell populations responding to bacterial infection. J Immunol. (2000) 165:6833–9. 10.4049/jimmunol.165.12.6833 11120806

[4] Badovinac VP Porter BB Harty JT. CD8+ T cell contraction is controlled by early inflammation. Nat Immunol. (2004) 5:809–17. 10.1038/ni1098 15247915

[5] Mescher MF Curtsinger JM Agarwal P Casey KA Gerner M Hammerbeck CD. Signals required for programming effector and memory development by CD8+ T cells. Immunol Rev. (2006) 211:81–92. 10.1111/j.0105-2896.2006.00382.x 16824119

[6] Banchereau J Palucka AK. Dendritic cells as therapeutic vaccines against cancer. Nat Rev Immunol. (2005) 5:296–306. 10.1038/nri1592 15803149

[7] Gutjahr A Tiraby G Perouzel E Verrier B Paul S. Triggering intracellular receptors for vaccine adjuvantation. Trends Immunol. (2016) 37:573–87. 10.1016/j.it.2016.07.001 27474233

[8] Nicoli F Finessi V Sicurella M Rizzotto L Gallerani E Destro F. The HIV-1 Tat protein induces the activation of CD8(+) T cells and affects *in vivo* the magnitude and kinetics of antiviral responses. PLoS ONE (2013) 8:e77746. 10.1371/journal.pone.0077746 24223723 PMC3817196

[9] Pavot V Rochereau N Resseguier J Gutjahr A Genin C Tiraby G. Cutting edge: new chimeric NOD2/TLR2 adjuvant drastically increases vaccine immunogenicity. J Immunol. (2014) 193:5781–5. 10.4049/jimmunol.1402184 25392526

[10] Kawai T Akira S. The role of pattern-recognition receptors in innate immunity: update on Toll-like receptors. Nat Immunol. (2010) 11:373–84. 10.1038/ni.1863 20404851

[11] Coffman RL Sher A Seder RA. Vaccine adjuvants: putting innate immunity to work. Immunity (2010) 33:492–503. 10.1016/j.immuni.2010.10.002 21029960 PMC3420356

[12] Zhang L Romero P. Metabolic control of CD8(+) T cell fate decisions and antitumor immunity. Trends Mol Med. (2018) 24:30–48. 10.1016/j.molmed.2017.11.005 29246759

[13] Ron-Harel N Santos D Ghergurovich JM Sage PT Reddy A Lovitch SB. Mitochondrial biogenesis and proteome remodeling promote one-carbon metabolism for T cell activation. Cell Metab. (2016) 24:104–17. 10.1016/j.cmet.2016.06.007 27411012 PMC5330619

[14] van der Windt GJ O'Sullivan D Everts B Huang SC Buck MD Curtis JD. CD8 memory T cells have a bioenergetic advantage that underlies their rapid recall ability. Proc Natl Acad Sci USA. (2013) 110:14336–41. 10.1073/pnas.1221740110 23940348 PMC3761631

[15] Almeida L Lochner M Berod L Sparwasser T. Metabolic pathways in T cell activation and lineage differentiation. Semin Immunol. (2016) 28:514–24. 10.1016/j.smim.2016.10.009 27825556

[16] van der Windt GJ Pearce EL. Metabolic switching and fuel choice during T-cell differentiation and memory development. Immunol Rev. (2012) 249:27–42. 10.1111/j.1600-065X.2012.01150.x 22889213 PMC3645891

[17] Cham CM Driessens G O'Keefe JP Gajewski TF. Glucose deprivation inhibits multiple key gene expression events and effector functions in CD8+ T cells. Eur J Immunol. (2008) 38:2438–50. 10.1002/eji.200838289 18792400 PMC3008428

[18] Wang R Dillon CP Shi LZ Milasta S Carter R Finkelstein D. The transcription factor Myc controls metabolic reprogramming upon T lymphocyte activation. Immunity (2011) 35:871–82. 10.1016/j.immuni.2011.09.021 22195744 PMC3248798

[19] O'Sullivan D van der Windt GJ Huang SC Curtis JD Chang CH Buck MD. Memory CD8(+) T cells use cell-intrinsic lipolysis to support the metabolic programming necessary for development. Immunity (2014) 41:75–88. 10.1016/j.immuni.2014.06.005 25001241 PMC4120664

[20] Shehata HM Murphy AJ Lee MKS Gardiner CM Crowe SM Sanjabi S. Sugar or fat?-metabolic requirements for immunity to viral infections. Front Immunol. (2017) 8:1311. 10.3389/fimmu.2017.01311 29085369 PMC5649203

[21] Pearce EJ Everts B. Dendritic cell metabolism. Nat Rev Immunol. (2015) 15:18–29. 10.1038/nri3771 25534620 PMC4495583

[22] Krawczyk CM Holowka T Sun J Blagih J Amiel E DeBerardinis RJ. Toll-like receptor–induced changes in glycolytic metabolism regulate dendritic cell activation. Blood (2010) 115:4742–9. 10.1182/blood-2009-10-249540 20351312 PMC2890190

[23] Everts B Amiel E Huang SC Smith AM Chang CH Lam WY. TLR-driven early glycolytic reprogramming via the kinases TBK1-IKKvarepsilon supports the anabolic demands of dendritic cell activation. Nat Immunol. (2014) 15:323–32. 10.1038/ni.2833 24562310 PMC4358322

[24] Dominguez-Andres J Arts RJW Ter Horst R Gresnigt MS Smeekens SP Ratter JM. Rewiring monocyte glucose metabolism via C-type lectin signaling protects against disseminated candidiasis. PLoS Pathog. (2017) 13:e1006632. 10.1371/journal.ppat.1006632 28922415 PMC5619837

[25] Saas P Varin A Perruche S Ceroi A. Recent insights into the implications of metabolism in plasmacytoid dendritic cell innate functions: potential ways to control these functions. F1000Res. (2017) 6:456. 10.12688/f1000research.11332.1 28580131 PMC5437952

[26] Michalek RD Gerriets VA Jacobs SR Macintyre AN MacIver NJ Mason EF. Cutting edge: distinct glycolytic and lipid oxidative metabolic programs are essential for effector and regulatory CD4+ T cell subsets. J Immunol. (2011) 186:3299–303. 10.4049/jimmunol.1003613 21317389 PMC3198034

[27] Menk AV Scharping NE Moreci RS Zeng X Guy C Salvatore S. Early TCR signaling induces rapid aerobic glycolysis enabling distinct acute T cell effector functions. Cell Rep. (2018) 22:1509–21. 10.1016/j.celrep.2018.01.040 29425506 PMC5973810

[28] Delgoffe GM Kole TP Zheng Y Zarek PE Matthews KL Xiao B. The mTOR kinase differentially regulates effector and regulatory T cell lineage commitment. Immunity (2009) 30:832–44. 10.1016/j.immuni.2009.04.014 19538929 PMC2768135

[29] Araki K Turner AP Shaffer VO Gangappa S Keller SA Bachmann MF. mTOR regulates memory CD8 T-cell differentiation. Nature (2009) 460:108–12. 10.1038/nature08155 19543266 PMC2710807

[30] Woltman AM van der Kooij SW Coffer PJ Offringa R Daha MR van Kooten C. Rapamycin specifically interferes with GM-CSF signaling in human dendritic cells, leading to apoptosis via increased p27KIP1 expression. Blood (2003) 101:1439–45. 10.1182/blood-2002-06-1688 12393532

[31] Hackstein H Taner T Zahorchak AF Morelli AE Logar AJ Gessner A. Rapamycin inhibits IL-4–induced dendritic cell maturation *in vitro* and dendritic cell mobilization and function *in vivo*. Blood (2003) 101:4457–63. 10.1182/blood-2002-11-3370 12531798

[32] Monti P Mercalli A Leone BE Valerio DC Allavena P Piemonti L. Rapamycin impairs antigen uptake of human dendritic cells. Transplantation (2003) 75:137–45. 10.1097/00007890-200301150-00025 12544886

[33] Haidinger M Poglitsch M Geyeregger R Kasturi S Zeyda M Zlabinger GJ. A versatile role of mammalian target of rapamycin in human dendritic cell function and differentiation. J Immunol. (2010) 185:3919–31. 10.4049/jimmunol.1000296 20805416

[34] Yang CS Song CH Lee JS Jung SB Oh JH Park J. Intracellular network of phosphatidylinositol 3-kinase, mammalian target of the rapamycin/70 kDa ribosomal S6 kinase 1, and mitogen-activated protein kinases pathways for regulating mycobacteria-induced IL-23 expression in human macrophages. Cell Microbiol. (2006) 8:1158–71. 10.1111/j.1462-5822.2006.00699.x 16819968

[35] Land SC Tee AR. Hypoxia-inducible factor 1alpha is regulated by the mammalian target of rapamycin (mTOR) via an mTOR signaling motif. J Biol Chem. (2007) 282:20534–43. 10.1074/jbc.M611782200 17502379

[36] Finlay DK Rosenzweig E Sinclair LV Feijoo-Carnero C Hukelmann JL Rolf J. PDK1 regulation of mTOR and hypoxia-inducible factor 1 integrate metabolism and migration of CD8+ T cells. J Exp Med. (2012) 209:2441–53. 10.1084/jem.20112607 23183047 PMC3526360

[37] Cheng SC Quintin J Cramer RA Shepardson KM Saeed S Kumar V. mTOR- and HIF-1alpha-mediated aerobic glycolysis as metabolic basis for trained immunity. Science (2014) 345:1250684. 10.1126/science.1250684 25258083 PMC4226238

[38] Ohtani M Nagai S Kondo S Mizuno S Nakamura K Tanabe M. Mammalian target of rapamycin and glycogen synthase kinase 3 differentially regulate lipopolysaccharide-induced interleukin-12 production in dendritic cells. Blood (2008) 112:635–43. 10.1182/blood-2008-02-137430 18492954 PMC2481549

[39] Weichhart T Werzowa J Horl WH Saemann MD. Biological action of rapamycin in renal transplantation. Am J Kidney Dis. (2008) 51:531; author reply 531–2. 10.1053/j.ajkd.2007.09.027 18295073

[40] Kidani Y Elsaesser H Hock MB Vergnes L Williams KJ Argus JP. Sterol regulatory element-binding proteins are essential for the metabolic programming of effector T cells and adaptive immunity. Nat Immunol. (2013) 14:489–99. 10.1038/ni.2570 23563690 PMC3652626

[41] Angela M Endo Y Asou HK Yamamoto T Tumes DJ Tokuyama H. Fatty acid metabolic reprogramming via mTOR-mediated inductions of PPARgamma directs early activation of T cells. Nat Commun. (2016) 7:13683. 10.1038/ncomms13683 27901044 PMC5141517

[42] Ibrahim J Nguyen AH Rehman A Ochi A Jamal M Graffeo CS. Dendritic cell populations with different concentrations of lipid regulate tolerance and immunity in mouse and human liver. Gastroenterology (2012) 143:1061–72. 10.1053/j.gastro.2012.06.003 22705178 PMC3459067

[43] Herber DL Cao W Nefedova Y Novitskiy SV Nagaraj S Tyurin VA. Lipid accumulation and dendritic cell dysfunction in cancer. Nat Med. (2010) 16:880–6. 10.1038/nm.2172 20622859 PMC2917488

[44] de Jong AJ Kloppenburg M Toes RE Ioan-Facsinay A. Fatty acids, lipid mediators, and T-cell function. Front Immunol. (2014) 5:483. 10.3389/fimmu.2014.00483 25352844 PMC4195378

[45] Al-Saffar NM Titley JC Robertson D Clarke PA Jackson LE Leach MO. Apoptosis is associated with triacylglycerol accumulation in Jurkat T-cells. Br J Cancer (2002) 86:963–70. 10.1038/sj.bjc.6600188 11953830 PMC2364152

[46] Gregg KA Harberts E Gardner FM Pelletier MR Cayatte C Yu L. Rationally designed TLR4 ligands for vaccine adjuvant discovery. MBio (2017) 8:e00492–17. 10.1128/mBio.00492-17 28487429 PMC5424205

[47] Park SN Noh KT Jeong YI Jung ID Kang HK Cha GS. Rhamnogalacturonan II is a Toll-like receptor 4 agonist that inhibits tumor growth by activating dendritic cell-mediated CD8+ T cells. Exp Mol Med. (2013) 45:e8. 10.1038/emm.2013.14 23392255 PMC3584663

[48] Reed SG Hsu FC Carter D Orr MT. The science of vaccine adjuvants: advances in TLR4 ligand adjuvants. Curr Opin Immunol. (2016) 41:85–90. 10.1016/j.coi.2016.06.007 27392183

[49] Rogero MM Calder PC. Obesity, inflammation, toll-like receptor 4 and fatty acids. Nutrients (2018) 10:E432. 10.3390/nu10040432 29601492 PMC5946217

[50] Shi H Kokoeva MV Inouye K Tzameli I Yin H Flier JS. TLR4 links innate immunity and fatty acid-induced insulin resistance. J Clin Invest. (2006) 116:3015–25. 10.1172/JCI28898 17053832 PMC1616196

[51] Ding Y Subramanian S Montes VN Goodspeed L Wang S Han C. Toll-like receptor 4 deficiency decreases atherosclerosis but does not protect against inflammation in obese low-density lipoprotein receptor-deficient mice. Arterioscler Thromb Vasc Biol. (2012) 32:1596–604. 10.1161/ATVBAHA.112.249847 22580897 PMC3748807

[52] Kim F Pham M Luttrell I Bannerman DD Tupper J Thaler J. Toll-like receptor-4 mediates vascular inflammation and insulin resistance in diet-induced obesity. Circ Res. (2007) 100:1589–96. 10.1161/CIRCRESAHA.106.142851 17478729

[53] Wang Y Qian Y Fang Q Zhong P Li W Wang L. Saturated palmitic acid induces myocardial inflammatory injuries through direct binding to TLR4 accessory protein MD2. Nat Commun. (2017) 8:13997. 10.1038/ncomms13997 28045026 PMC5216130

[54] Lee JJ Wang PW Yang IH Huang HM Chang CS Wu CL. High-fat diet induces toll-like receptor 4-dependent macrophage/microglial cell activation and retinal impairment. Invest Ophthalmol Vis Sci. (2015) 56:3041–50. 10.1167/iovs.15-16504 26024088

[55] Lancaster GI Langley KG Berglund NA Kammoun HL Reibe S Estevez E. Evidence that TLR4 is not a receptor for saturated fatty acids but mediates lipid-induced inflammation by reprogramming macrophage metabolism. Cell Metab. (2018) 27:1096–110.e5. 10.1016/j.cmet.2018.03.014 29681442

[56] Rocha DM Caldas AP Oliveira LL Bressan J Hermsdorff HH. Saturated fatty acids trigger TLR4-mediated inflammatory response. Atherosclerosis (2016) 244:211–5. 10.1016/j.atherosclerosis.2015.11.015 26687466

[57] Steimle A Autenrieth IB Frick JS. Structure and function: lipid A modifications in commensals and pathogens. Int J Med Microbiol. (2016) 306:290–301. 10.1016/j.ijmm.2016.03.001 27009633

[58] Lee JY Ye J Gao Z Youn HS Lee WH Zhao L. Reciprocal modulation of Toll-like receptor-4 signaling pathways involving MyD88 and phosphatidylinositol 3-kinase/AKT by saturated and polyunsaturated fatty acids. J Biol Chem. (2003) 278:37041–51. 10.1074/jbc.M305213200 12865424

[59] Lee JY Zhao L Youn HS Weatherill AR Tapping R Feng L. Saturated fatty acid activates but polyunsaturated fatty acid inhibits Toll-like receptor 2 dimerized with Toll-like receptor 6 or 1. J Biol Chem. (2004) 279:16971–9. 10.1074/jbc.M312990200 14966134

[60] Hwang DH Kim JA Lee JY. Mechanisms for the activation of Toll-like receptor 2/4 by saturated fatty acids and inhibition by docosahexaenoic acid. Eur J Pharmacol. (2016) 785:24–35. 10.1016/j.ejphar.2016.04.024 27085899 PMC5815395

[61] Wong SW Kwon MJ Choi AM Kim HP Nakahira K Hwang DH. Fatty acids modulate Toll-like receptor 4 activation through regulation of receptor dimerization and recruitment into lipid rafts in a reactive oxygen species-dependent manner. J Biol Chem. (2009) 284:27384–92. 10.1074/jbc.M109.044065 19648648 PMC2785667

[62] Weatherill AR Lee JY Zhao L Lemay DG Youn HS Hwang DH. Saturated and polyunsaturated fatty acids reciprocally modulate dendritic cell functions mediated through TLR4. J Immunol. (2005) 174:5390–7. 10.4049/jimmunol.174.9.5390 15843537

[63] Carlsson JA Wold AE Sandberg AS Ostman SM. The polyunsaturated fatty acids arachidonic acid and docosahexaenoic acid induce mouse dendritic cells maturation but reduce T-cell responses *in vitro*. PLoS ONE (2015) 10:e0143741. 10.1371/journal.pone.0143741 26619195 PMC4664484

[64] Brix S Lund P Kjaer TM Straarup EM Hellgren LI Frokiaer H. CD4(+) T-cell activation is differentially modulated by bacteria-primed dendritic cells, but is generally down-regulated by n-3 polyunsaturated fatty acids. Immunology (2010) 129:338–50. 10.1111/j.1365-2567.2009.03163.x 19909377 PMC2826679

[65] McMurray DN Jolly CA Chapkin RS. Effects of dietary n-3 fatty acids on T cell activation and T cell receptor-mediated signaling in a murine model. J Infect Dis. (2000) 182(Suppl. 1):S103–7. 10.1086/315909 10944491

[66] Oh DY Talukdar S Bae EJ Imamura T Morinaga H Fan W. GPR120 is an omega-3 fatty acid receptor mediating potent anti-inflammatory and insulin-sensitizing effects. Cell (2010) 142:687–98. 10.1016/j.cell.2010.07.041 20813258 PMC2956412

[67] Stulnig TM Huber J Leitinger N Imre EM Angelisova P Nowotny P. Polyunsaturated eicosapentaenoic acid displaces proteins from membrane rafts by altering raft lipid composition. J Biol Chem. (2001) 276:37335–40. 10.1074/jbc.M106193200 11489905

[68] Fan YY Ly LH Barhoumi R McMurray DN Chapkin RS. Dietary docosahexaenoic acid suppresses T cell protein kinase C theta lipid raft recruitment and IL-2 production. J Immunol. (2004) 173:6151–60. 10.4049/jimmunol.173.10.6151 15528352

[69] Chapkin RS Arrington JL Apanasovich TV Carroll RJ McMurray DN. Dietary n-3 PUFA affect TcR-mediated activation of purified murine T cells and accessory cell function in co-cultures. Clin Exp Immunol. (2002) 130:12–8. 10.1046/j.1365-2249.2002.01951.x 12296847 PMC1906501

[70] Zurier RB Rossetti RG Seiler CM Laposata M. Human peripheral blood T lymphocyte proliferation after activation of the T cell receptor: effects of unsaturated fatty acids. Prostaglandins Leukot Essent Fatty Acids (1999) 60:371–5. 10.1016/S0952-3278(99)80015-5 10471124

[71] Shaikh SR Boyle S Edidin M. A high fat diet containing saturated but not unsaturated fatty acids enhances T cell receptor clustering on the nanoscale. Prostaglandins Leukot Essent Fatty Acids (2015) 100:1–4. 10.1016/j.plefa.2015.05.001 26143085 PMC4554807

[72] Lee J Walsh MC Hoehn KL James DE Wherry EJ Choi Y. Regulator of fatty acid metabolism, acetyl coenzyme a carboxylase 1, controls T cell immunity. J Immunol. (2014) 192:3190–9. 10.4049/jimmunol.1302985 24567531 PMC3965631

[73] Shaikh SR Mitchell D Carroll E Li M Schneck J Edidin M. Differential effects of a saturated and a monounsaturated fatty acid on MHC class I antigen presentation. Scand J Immunol. (2008) 68:30–42. 10.1111/j.1365-3083.2008.02113.x 18533931 PMC2805012

[74] Pearce EL Walsh MC Cejas PJ Harms GM Shen H Wang LS. Enhancing CD8 T-cell memory by modulating fatty acid metabolism. Nature (2009) 460:103–7. 10.1038/nature08097 19494812 PMC2803086

[75] Raulien N Friedrich K Strobel S Rubner S Baumann S von Bergen M. Fatty acid oxidation compensates for lipopolysaccharide-induced warburg effect in glucose-deprived monocytes. Front Immunol. (2017) 8:609. 10.3389/fimmu.2017.00609 28611773 PMC5447039

[76] Zhang Y Kurupati R Liu L Zhou XY Zhang G Hudaihed A. Enhancing CD8+ T cell fatty acid catabolism within a metabolically challenging tumor microenvironment increases the efficacy of melanoma immunotherapy. Cancer Cell (2017) 32:377–91.e9. 10.1016/j.ccell.2017.08.004 28898698 PMC5751418

[77] Byersdorfer CA Tkachev V Opipari AW Goodell S Swanson J Sandquist S. Effector T cells require fatty acid metabolism during murine graft-versus-host disease. Blood (2013) 122:3230–7. 10.1182/blood-2013-04-495515 24046012 PMC3814737

[78] Desmet CJ Ishii KJ. Nucleic acid sensing at the interface between innate and adaptive immunity in vaccination. Nat Rev Immunol. (2012) 12:479–91. 10.1038/nri3247 22728526

[79] Gowda NM Wu X Gowda DC. TLR9 and MyD88 are crucial for the development of protective immunity to malaria. J Immunol. (2012) 188:5073–85. 10.4049/jimmunol.1102143 22516959 PMC3345097

[80] Ives A Masina S Castiglioni P Prevel F Revaz-Breton M Hartley MA. MyD88 and TLR9 dependent immune responses mediate resistance to Leishmania guyanensis infections, irrespective of Leishmania RNA virus burden. PLoS ONE (2014) 9:e96766. 10.1371/journal.pone.0096766 24801628 PMC4011865

[81] Scheiermann J Klinman DM. Clinical evaluation of CpG oligonucleotides as adjuvants for vaccines targeting infectious diseases and cancer. Vaccine (2014) 32:6377–89. 10.1016/j.vaccine.2014.06.065 24975812 PMC4252359

[82] Tudor D Dubuquoy C Gaboriau V Lefevre F Charley B Riffault S. TLR9 pathway is involved in adjuvant effects of plasmid DNA-based vaccines. Vaccine (2005) 23:1258–64. 10.1016/j.vaccine.2004.09.001 15652668

[83] Fallarino F Volpi C Zelante T Vacca C Calvitti M Fioretti MC. IDO mediates TLR9-driven protection from experimental autoimmune diabetes. J Immunol. (2009) 183:6303–12. 10.4049/jimmunol.0901577 19841163

[84] Ciorba MA Bettonville EE McDonald KG Metz R Prendergast GC Newberry RD. Induction of IDO-1 by immunostimulatory DNA limits severity of experimental colitis. J Immunol. (2010) 184:3907–16. 10.4049/jimmunol.0900291 20181893 PMC2945286

[85] Wang YZ Lv H Hao YL Zhang HQ Li L Cai GM. Suppressive oligodeoxynucleotides induced tolerogenic plasmacytoid dendritic cells and ameliorated the experimental autoimmune neuritis. Immunol Lett. (2015) 166:13–8. 10.1016/j.imlet.2015.04.007 25952624

[86] Wingender G Garbi N Schumak B Jungerkes F Endl E von Bubnoff D. Systemic application of CpG-rich DNA suppresses adaptive T cell immunity via induction of IDO. Eur J Immunol. (2006) 36:12–20. 10.1002/eji.200535602 16323249

[87] Xin L Shelite TR Gong B Mendell NL Soong L Fang R. Systemic treatment with CpG-B after sublethal rickettsial infection induces mouse death through indoleamine 2,3-dioxygenase (IDO). PLoS ONE (2012) 7:e34062. 10.1371/journal.pone.0034062 22470514 PMC3314704

[88] Mellor AL Baban B Chandler PR Manlapat A Kahler DJ Munn DH. Cutting edge: CpG oligonucleotides induce splenic CD19+ dendritic cells to acquire potent indoleamine 2,3-dioxygenase-dependent T cell regulatory functions via IFN Type 1 signaling. J Immunol. (2005) 175:5601–5. 10.4049/jimmunol.175.9.5601 16237046

[89] Baban B Chandler PR Sharma MD Pihkala J Koni PA Munn DH. IDO activates regulatory T cells and blocks their conversion into Th17-like T cells. J Immunol. (2009) 183:2475–83. 10.4049/jimmunol.0900986 19635913 PMC3677163

[90] Ehrlich AK Fernandez OL Rodriguez-Pinto D Castilho TM Corral Caridad MJ Goldsmith-Pestana K. Local delivery of the toll-like receptor 9 ligand CpG downregulates host immune and inflammatory responses, ameliorating established *Leishmania* (viannia) panamensis chronic infection. Infect Immun. (2017) 85:e00981–16. 10.1128/IAI.00981-16 28052994 PMC5328479

[91] Chen YB Li SD He YP Shi XJ Chen Y Gong JP. Immunosuppressive effect of IDO on T cells in patients with chronic hepatitis B^*^. Hepatol Res. (2009) 39:463–8. 10.1111/j.1872-034X.2008.00476.x 19207575

[92] Schmidt SV Schultze JL. New Insights into IDO biology in bacterial and viral infections. Front Immunol. (2014) 5:384. 10.3389/fimmu.2014.00384 25157255 PMC4128074

[93] Routy JP Routy B Graziani GM Mehraj V. The kynurenine pathway is a double-edged sword in immune-privileged sites and in cancer: implications for immunotherapy. Int J Tryptophan Res. (2016) 9:67–77. 10.4137/IJTR.S38355 27773992 PMC5063567

[94] Munn DH Shafizadeh E Attwood JT Bondarev I Pashine A Mellor AL. Inhibition of T cell proliferation by macrophage tryptophan catabolism. J Exp Med. (1999) 189:1363–72. 10.1084/jem.189.9.1363 10224276 PMC2193062

[95] Chung DJ Rossi M Romano E Ghith J Yuan J Munn DH. Indoleamine 2,3-dioxygenase-expressing mature human monocyte-derived dendritic cells expand potent autologous regulatory T cells. Blood (2009) 114:555–63. 10.1182/blood-2008-11-191197 19465693 PMC2713474

[96] Lippens C Duraes FV Dubrot J Brighouse D Lacroix M Irla M. IDO-orchestrated crosstalk between pDCs and Tregs inhibits autoimmunity. J Autoimmun. (2016) 75:39–49. 10.1016/j.jaut.2016.07.004 27470005 PMC5127883

[97] Sharma MD Hou DY Baban B Koni PA He Y Chandler PR. Reprogrammed foxp3(+) regulatory T cells provide essential help to support cross-presentation and CD8(+) T cell priming in naive mice. Immunity (2010) 33:942–54. 10.1016/j.immuni.2010.11.022 21145762 PMC3032429

[98] Fox JM Sage LK Huang L Barber J Klonowski KD Mellor AL. Inhibition of indoleamine 2,3-dioxygenase enhances the T-cell response to influenza virus infection. J Gen Virol. (2013) 94:1451–61. 10.1099/vir.0.053124-0 23580425 PMC3709631

[99] Sage LK Fox JM Mellor AL Tompkins SM Tripp RA. Indoleamine 2,3-dioxygenase (IDO) activity during the primary immune response to influenza infection modifies the memory T cell response to influenza challenge. Viral Immunol. (2014) 27:112–23. 10.1089/vim.2013.0105 24702331 PMC3995207

[100] Barber GN. STING: infection, inflammation and cancer. Nat Rev Immunol. (2015) 15:760–70. 10.1038/nri3921 26603901 PMC5004891

[101] Chen Q Sun L Chen ZJ. Regulation and function of the cGAS-STING pathway of cytosolic DNA sensing. Nat Immunol. (2016) 17:1142–9. 10.1038/ni.3558 27648547

[102] Iurescia S Fioretti D Rinaldi M. Nucleic acid sensing machinery: targeting innate immune system for cancer therapy. Recent Pat Anticancer Drug Discov. (2018) 13:2–17. 10.2174/1574892812666171030163804 29086701

[103] Christensen MH Paludan SR. Viral evasion of DNA-stimulated innate immune responses. Cell Mol Immunol. (2017) 14:4–13. 10.1038/cmi.2016.06 26972769 PMC5214947

[104] Junt T Barchet W. Translating nucleic acid-sensing pathways into therapies. Nat Rev Immunol. (2015) 15:529–44. 10.1038/nri3875 26292638

[105] Dey B Dey RJ Cheung LS Pokkali S Guo H Lee JH. A bacterial cyclic dinucleotide activates the cytosolic surveillance pathway and mediates innate resistance to tuberculosis. Nat Med. (2015) 21:401–6. 10.1038/nm.3813 25730264 PMC4390473

[106] Collins AC Cai H Li T Franco LH Li XD Nair VR. Cyclic GMP-AMP synthase is an innate immune DNA sensor for *Mycobacterium tuberculosis*. Cell Host Microbe (2015) 17:820–8. 10.1016/j.chom.2015.05.005 26048137 PMC4499468

[107] Rasmussen SB Horan KA Holm CK Stranks AJ Mettenleiter TC Simon AK. Activation of autophagy by alpha-herpesviruses in myeloid cells is mediated by cytoplasmic viral DNA through a mechanism dependent on stimulator of IFN genes. J Immunol. (2011) 187:5268–76. 10.4049/jimmunol.1100949 21998456 PMC3208073

[108] Saitoh T Fujita N Hayashi T Takahara K Satoh T Lee H. Atg9a controls dsDNA-driven dynamic translocation of STING and the innate immune response. Proc Natl Acad Sci USA. (2009) 106:20842–6. 10.1073/pnas.0911267106 19926846 PMC2791563

[109] Li Y Wilson HL Kiss-Toth E. Regulating STING in health and disease. J Inflamm. (2017) 14:11. 10.1186/s12950-017-0159-2 28596706 PMC5463399

[110] Konno H Konno K Barber GN. Cyclic dinucleotides trigger ULK1 (ATG1) phosphorylation of STING to prevent sustained innate immune signaling. Cell (2013) 155:688–98. 10.1016/j.cell.2013.09.049 24119841 PMC3881181

[111] Stjepanovic G Baskaran S Lin MG Hurley JH. Vps34 kinase domain dynamics regulate the autophagic PI 3-kinase complex. Mol Cell (2017) 67:528–34.e3. 10.1016/j.molcel.2017.07.003 28757208 PMC5573195

[112] Liang Q Seo GJ Choi YJ Kwak MJ Ge J Rodgers MA. Crosstalk between the cGAS DNA sensor and Beclin-1 autophagy protein shapes innate antimicrobial immune responses. Cell Host Microbe (2014) 15:228–38. 10.1016/j.chom.2014.01.009 24528868 PMC3950946

[113] Prabakaran T Bodda C Krapp C Zhang BC Christensen MH Sun C. Attenuation of cGAS-STING signaling is mediated by a p62/SQSTM1-dependent autophagy pathway activated by TBK1. EMBO J. (2018) 37:e97858. 10.15252/embj.201797858 29496741 PMC5897779

[114] Criollo A Chereau F Malik SA Niso-Santano M Marino G Galluzzi L. Autophagy is required for the activation of NFkappaB. Cell Cycle (2012) 11:194–9. 10.4161/cc.11.1.18669 22186785

[115] Paul S Kashyap AK Jia W He YW Schaefer BC. Selective autophagy of the adaptor protein Bcl10 modulates T cell receptor activation of NF-kappaB. Immunity (2012) 36:947–58. 10.1016/j.immuni.2012.04.008 22658522 PMC3389288

[116] Whang MI Tavares RM Benjamin DI Kattah MG Advincula R Nomura DK. The ubiquitin binding protein TAX1BP1 mediates autophagasome induction and the metabolic transition of activated T cells. Immunity (2017) 46:405–20. 10.1016/j.immuni.2017.02.018 28314591 PMC5400745

[117] Botbol Y Guerrero-Ros I Macian F. Key roles of autophagy in regulating T-cell function. Eur J Immunol. (2016) 46:1326–34. 10.1002/eji.201545955 27151577 PMC5227655

[118] Puleston DJ Zhang H Powell TJ Lipina E Sims S Panse I. Autophagy is a critical regulator of memory CD8(+) T cell formation. Elife (2014) 3:1–21. 10.7554/eLife.03706 25385531 PMC4225493

[119] Xu X Araki K Li S Han JH Ye L Tan WG. Autophagy is essential for effector CD8(+) T cell survival and memory formation. Nat Immunol. (2014) 15:1152–61. 10.1038/ni.3025 25362489 PMC4232981

[120] Prantner D Perkins DJ Vogel SN. AMP-activated Kinase (AMPK) promotes innate immunity and antiviral defense through modulation of stimulator of interferon genes (STING) signaling. J Biol Chem. (2017) 292:292–304. 10.1074/jbc.M116.763268 27879319 PMC5217687

[121] Ma EH Poffenberger MC Wong AH Jones RG. The role of AMPK in T cell metabolism and function. Curr Opin Immunol. (2017) 46:45–52. 10.1016/j.coi.2017.04.004 28460345

[122] Blagih J Coulombe F Vincent EE Dupuy F Galicia-Vazquez G Yurchenko E. The energy sensor AMPK regulates T cell metabolic adaptation and effector responses *in vivo*. Immunity (2015) 42:41–54. 10.1016/j.immuni.2014.12.030 25607458

[123] Eikawa S Nishida M Mizukami S Yamazaki C Nakayama E Udono H. Immune-mediated antitumor effect by type 2 diabetes drug, metformin. Proc Natl Acad Sci USA. (2015) 112:1809–14. 10.1073/pnas.1417636112 25624476 PMC4330733

